# Toward a Conceptual Framework for Digitally Supported Communication, Coordination, Cooperation, and Collaboration in Interprofessional Health Care: Scoping Review

**DOI:** 10.2196/69276

**Published:** 2025-05-26

**Authors:** Kim Nordmann, Marie-Christin Redlich, Michael Schaller, Stefanie Sauter, Florian Fischer

**Affiliations:** 1 Bavarian Research Center for Digital Health and Social Care Kempten Germany

**Keywords:** care continuity, communication, collaboration, coordination, interprofessional relations

## Abstract

**Background:**

Digital tools for communication, coordination, cooperation, and collaboration (D4C), including electronic health records and specialized apps, are increasingly used in health care to ensure continuity of care across professional boundaries. Despite their growing adoption, there is a lack of precise and clear definitions, and no common understanding of D4C within health care.

**Objective:**

This study aims to explore the concepts and definitions of digitally supported communication, coordination, cooperation, and collaboration by mapping the individual attributes to build a foundation for the operationalization of these concepts and to generate a clear and precise understanding of these concepts in research, practice, and policy.

**Methods:**

A scoping review was conducted across MEDLINE, CINAHL, Embase, PsycINFO, and Scopus to identify studies on D4C. We included peer-reviewed studies in English, French, German, Portuguese, and Spanish published since 2012. Definitions of the modes of interaction (communication, coordination, cooperation, and collaboration) and the digital tool supporting these interactions, along with their definitions in cited references, were extracted and analyzed.

**Results:**

Of the 407 identified papers addressing D4C, 6.1% (n=25) defined the digital concept and 6.6% (n=27) defined the interaction supported by the digital tool, with even fewer being backed by a reference. The analysis of the definitions revealed a hierarchical framework, detailing dimensions, requisites, and goals for each mode of interaction and the digital tool. It delineates progression from communication to collaboration: communication enables the exchange of information; coordination involves organizing people, resources, and activities; cooperation focuses on dividing tasks to achieve shared goals; and collaboration, at the apex, involves jointly addressing care needs. Each mode of interaction can be supported by digital tools.

**Conclusions:**

The proposed framework offers a structured approach to establish a shared understanding of the concept of D4C. This unified understanding can serve as a foundation for developing objectives related to the implementation and evaluation of digital tools aimed at fostering interprofessional interactions in health care. As such, it can inform stakeholders in their understanding of D4C, possibly improving workflows and patient care. Further research is needed to operationalize and validate the framework across health care settings.

**International Registered Report Identifier (IRRID):**

RR2-10.2196/45179

## Introduction

Digital health tools, including telehealth, wearable devices, and health information technology, are on the rise. As of 2022, the global digital health market was estimated at US $211 billion, with projections suggesting an annual growth rate of 18.6% until 2030 [1]. These tools have become integral to health care services, being used in almost every aspect of health care routine, from prevention and detection to treatment and recovery, significantly improving patient outcomes [2].

Digital health tools have the potential to facilitate interaction between professionals in health care, enabling communication, coordination, cooperation, and collaboration (hereafter referred to as D4C tools) [3-6]. While communication refers to “a process by which information is exchanged between individuals through a common system of symbols, signs, or behavior” [7], the lines between the other three concepts are blurry [8]. According to the Merriam-Webster dictionary, coordination describes “the process of organizing people or groups so that they work together properly and well” [9], and cooperation refers to “the work and activity of a number of persons who individually contribute toward the efficiency of the whole” [10]. Collaboration is further defined as “the situation of two or more people working together to create or achieve the same thing” [11]. In the context of health care, examples of D4C tools include electronic medical records, telemonitoring systems. and web-based resources [12]. Their adoption is widespread, for example, 96% of general acute care clinics in the United States have implemented electronic health records [13]. The application of D4C tools is critical in the interprofessional and intersectoral context to ensure continuity of care across professional boundaries [3,4].

Despite the increasing reliance on D4C tools in health care, a comprehensive D4C framework is missing. Such a framework can serve as a guiding tool for establishing a shared understanding. It should support the differentiation between communication, coordination, cooperation, and collaboration processes, thereby helping to select implementation and evaluation strategies that are appropriate for the respective dimensions. Previous D4C models, such as the 3C Collaboration Model to guide the development of groupware and the Collaboration Space Model to support the development of technology for collaboration purposes, lack a nuanced framework on the distinctions and intersections of communication, coordination, cooperation, and collaboration to enhance design and evaluation of D4C tools [14,15]. This study aims to address this gap by developing such a comprehensive framework for interprofessional D4C, underpinned by an extensive scoping review. Our objective is to explore the concepts and definitions of digitally supported communication, coordination, cooperation, and collaboration by mapping the individual attributes to build a foundation for the operationalization and to generate a clear and precise understanding of these concepts in research, practice, and policy.

## Methods

### Overview

We conducted a scoping review to identify peer-reviewed papers on D4C used for interprofessional exchange among health care providers. The review followed the Joanna Briggs Institute methodology and a previously published review protocol [16,17]. The reporting adheres to the PRISMA-ScR (Preferred Reporting Items for Systematic Reviews and Meta-Analyses extension for Scoping Reviews; checklist provided in Multimedia Appendix 1) [18].

### Literature Sources and Selection

We applied a three-step approach to identify and include relevant papers. First, we fine-tuned our search string in PubMed by screening the titles of the search results obtained. To capture the entire breadth of publications in this subject area as a base for the framework, we used a sensitive search string (Table 1). Then, we adapted the search string to CINAHL, Embase, PsycINFO, and Scopus, checked for conclusiveness, and extracted the search results (Multimedia Appendix 2). After uploading all records to Covidence (Veritas Health Innovation, Melbourne, Australia), duplicates were eliminated. A preliminary screening of the first 50 records was conducted by all study authors. Screening results were discussed to ensure a uniform understanding of eligibility criteria. Each remaining abstract was screened independently by two researchers. Full-text screening was conducted for all included abstracts, and the studies were either included in the synthesis or excluded with a reason for exclusion. Discrepancies between researchers’ decisions were settled by an additional reviewer throughout all stages of the screening process. Lastly, KN manually screened the references of all included papers to identify additional studies.

**Table 1:** Search strategy on MEDLINE via PubMed and Scopus.

Participants, concept, and context and adaptation number			Search string		Hits in PubMed (December 11, 2022)		Hits in Scopus (December 11, 2022)
**Communication and collaboration among different health care provider groups**							
	1	trans-disciplin*^a^ OR transdisciplin*^a^ OR cross-disciplinar*^a^ OR crossdisciplinar*^a^ OR inter-disciplin*^a^ OR interdisciplin*^a^ OR multi-disciplin*^a^ OR multidisciplin*^a^ OR multi-profession*^a^ OR multiprofession*^a^ OR inter-profession*^a^ OR interprofession*^a^		185,543		392,636	
	2	“knowledge transfer”^a^ OR information*^a^ OR Health Information Exchange^b^ OR cooperat*^a^ OR co-operat*^a^ OR collaborat*^a^ OR communicat*^a^		2,089,524		7,334,822	
	3	“integrated care”^ a^ OR Intersectoral Collaboration^b^ OR Interdisciplinary Communication^b ^		26,343		33,558	
	4	(# 1 AND #2) OR 3#		75,493		144,245	
**Digital tools**							
	5	Health Information Systems^b^ OR Ambulatory Care Information Systems^b ^OR Information Technology^b^ OR technolog*^a^ OR socio-techni*^a^ OR sociotechni*^a^ OR mHealth^a^ OR eHealth^a^ OR digit*^a^ OR Electronic Health Records^b^ OR Public Health Informatics^b^ OR messag*^a^ OR messeng*^a^ OR app^a^ OR video*^a^ OR phone^a^ OR E-Mail*^a^ OR “E Mails”^a^ OR “E Mail”^a ^OR Email*^a^ OR “electronic mail”^a^ OR “electronic mails”^a^ OR “social media”^a ^OR WhatsApp^a ^OR Facebook^a^ OR Viber^a^ OR WeChat^a^ OR Telegram^a^ OR Kakotalk^a^		1,254,069		5,992,856	
**Health care setting**							
	6	Health*^a^ OR hospital*^a^ OR care*^a^ OR caring^a^		5,596,504		7,922,757	
**Combined**							
	7	#4 AND #5 AND #6		7261		11,056	
**Filters**							
	8	#7+English, French, Spanish, Portuguese, German, from 2012 onwards		5694		8216	

^a^Title or abstract.

^b^MeSH (Medical Subject Headings) term for PubMed search string and title or abstract or keyword for Scopus search string.

### Eligibility Criteria

The eligibility criteria encompassed formal attributes and aspects related to the content. In terms of formal attributes, we included any type of primary research approach and study design, opinion pieces, guidelines, reviews, meta-analyses, and meta-syntheses if they were published in a peer-reviewed journal and written in English, French, German, Portuguese, or Spanish. Conference abstracts, book chapters, and any records without access to the full text were excluded, as was anything published before 2012 due to the rapid development of digital technologies.

Regarding content, we included publications in any geographic and demographic health care setting focusing on D4C among at least two distinct groups of health care professionals or among health care professionals in similar roles but situated in different health care settings. We excluded studies that primarily investigated D4C between patient groups and health care practitioners, those examining the same health care profession within identical settings, and any focusing on students of health care professions. We further excluded papers with a focus on telemedicine—primarily facilitating interaction between health care providers and patients.

### Data Extraction and Analysis

All included records were imported into MAXQDA (version 2022; VERBI GmbH), and definitions of communication, coordination, cooperation, collaboration, and the digital tool in the papers were deductively coded. A definition hereby consisted of a definiendum (in this case communication, coordination, cooperation, collaboration, or the digital tool) and the definiens, that is, the explanation of the definiendum [19]. To derive the framework, we focused only on the definitions explicitly provided within the papers. Two researchers (MCR and KN) double-checked the coding of definitions and compiled a definitive list of the definitions and their corresponding references, using Microsoft Excel. All references for each definition were read and, if available, their definition extracted into the same sheet. If the cited references did not provide a definition, the definition of the paper was excluded from analysis. If we were unable to access or find the references, the definition was similarly excluded from the analysis. The extracted definitions of the original record and the reference were then processed by ChatGPT-3.5 (OpenAI) to generate ideas for prevailing themes and subthemes. Guided by these ideas, KN then inductively coded common themes and subthemes across all definitions, with the ultimate coding framework being reflected in the final framework presented in this paper: a characterization of each definition alongside corresponding attributes (ie, dimension, requisites, and goal).

### Ethical Considerations

No ethics approval was needed to conduct this scoping review of papers existing in the public domain.

## Results

### Search Results

**Figure 1:**
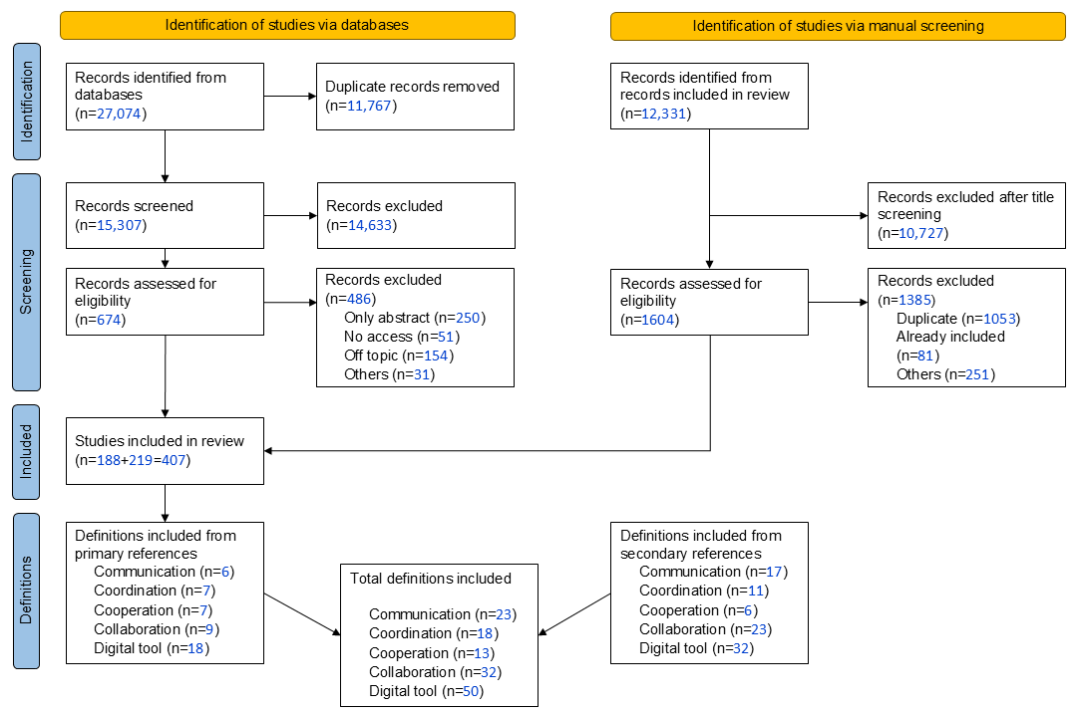
PRISMA flow diagram for study selection and derivation of definitions.

Through the database search, 27,074 papers were identified. The removal of 11,767 duplicates yielded 15,307 unique papers, of which 14,633 (95.6%) were excluded after title and abstract screening. Full-text review yielded a total of 188 (1.2%) included papers. Manually searching the 12,331 references led to the inclusion of 219 additional papers. The PRISMA (Preferred Reporting Items for Systematic Reviews and Meta-Analyses) flow diagram summarizes the process of inclusion and selection of papers and was extended by the manual addition of references (Figure 1) [18]. The weighted average of the two researchers’ Cohen κ at the abstract screening stage was 0.22 and for the full-text screening 0.53, indicating a fair interrater agreement for abstract screening and moderate agreement for full-text screening [20,21].

**Figure 2:**
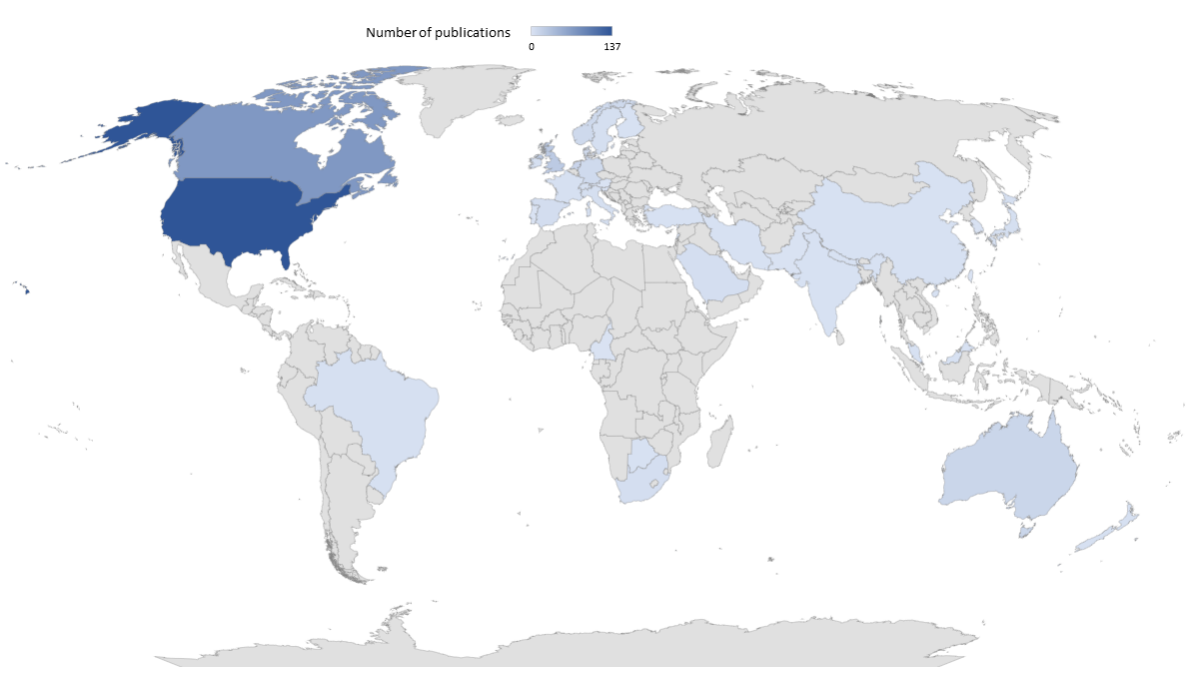
Country allocation of studies (n = 407); studies with multiple countries (n = 41) or without a country specification (n = 10) are excluded.

**Figure 3:**
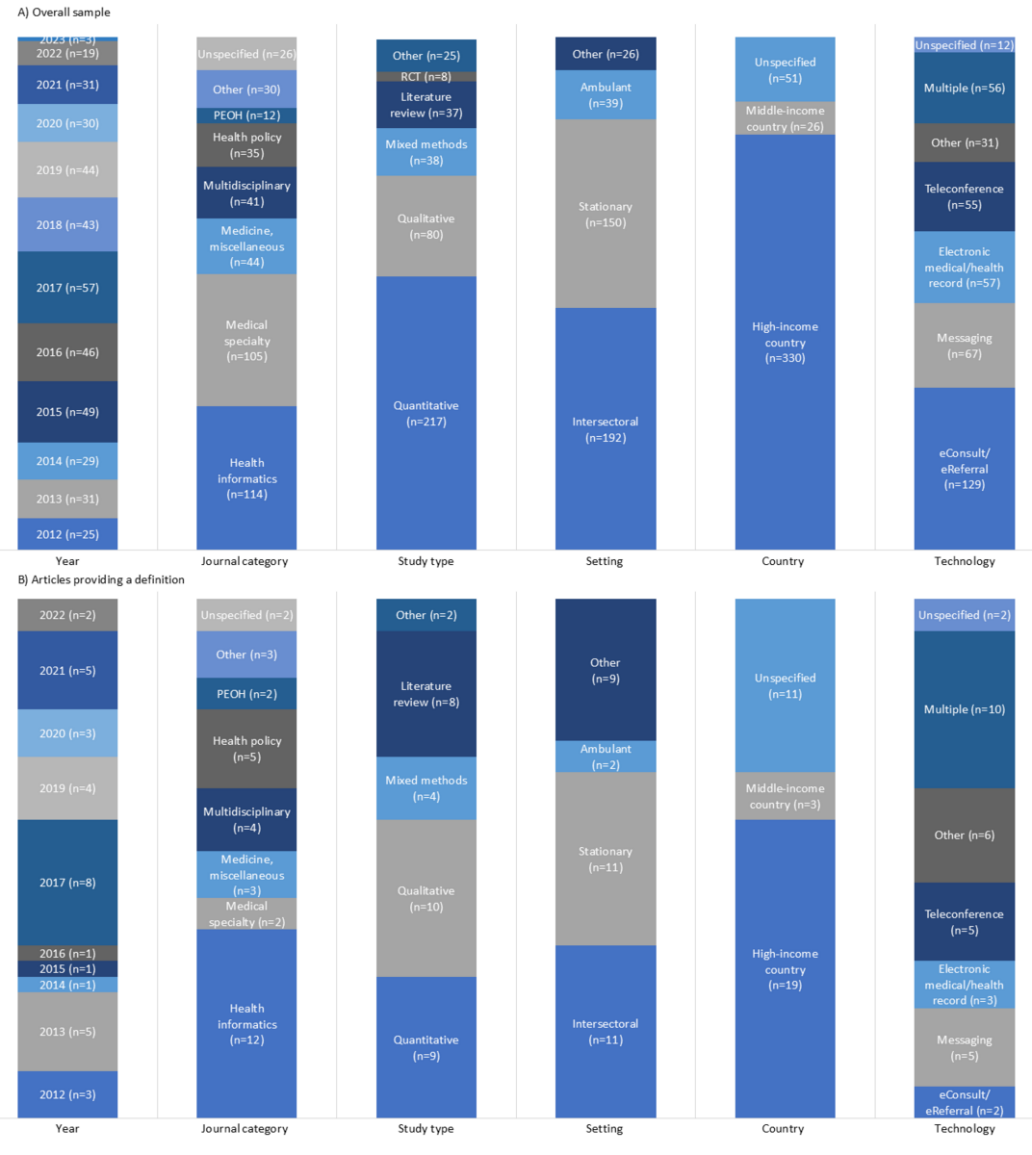
Comparison of the overall sample (n=407) and those papers which provided a definition (n=33). The unspecified group includes papers naming multiple countries or none. Messaging comprises instant messenger tools, email, pager, and SMS text messaging. Teleconference refers to hands-free communication devices, phone calls, teleconferences, and videoconferences. “Multiple” refers to studies reporting on various technologies. "Other” includes digital care pathways, virtual reality, health information exchange, social media, and medication management. PEOH: Public, Environmental, and Occupational Health; RCT: randomized controlled trial.

The scoping review identified 407 studies involving D4C tools. Precisely 81.1% (330) came from high-income countries, with the majority being from the United States (n=137) and Canada (n=72; Figure 2). The other studies were from middle-income countries (n=26), spanned over multiple countries (n=41), or did not specify a country (n=10). Most papers were published in journals in the subject area of medicine (n=349), with the main subject categories being health informatics (n=114) and medical specialties (n=105; Figure 3A). In total, 217 studies used a quantitative research approach, 80 used a qualitative research approach, and 38 studies were mixed-method studies. With regard to the settings in which digital health tools are used, 39 studies were ambulant, 150 examined hospitals, 192 were intersectoral, and the remaining 26 studies were not specific. The main technologies reported were eConsultations or eReferrals (n=129), messaging tools (n=67), electronic medical records or electronic health records (n=57), and teleconferences (n=55). More details for all papers are provided in Multimedia Appendix 3.

### Definitions

Of the 407 papers focusing on D4C tools, 33 papers were included in the final dataset for the analysis of definitions and derivation of the framework: 27 papers (6.6%) provided a total of 41 definitions of communication, coordination, cooperation, and/or collaboration. However, 12 (29.3%) definitions were excluded due to a lack of definition in the reference, resulting in: 6 definitions for communication [22-27], 7 each for coordination [24,25,27-31] and cooperation [24,25,27,30-33], and 9 for collaboration [26,27,31,33-38]. While many papers explained the D4C tool used in their study in detail, overarching technology was defined in only 25 papers (6.1%), of which 7 were excluded. Definitions were provided for: digital health (n=1) [39], eHealth (n=4) [27,40-42], health information exchange (n=2) [43,44], information and communication technologies (n=3) [26,45,46], mobile health (mHealth; n=2) [47,48], telehealth (n=1) [49], and telemedicine (n=5) [50-54]. Two references [26,27] gave definitions for (1) at least one concept of 4C and (2) at least one type of technology. Two papers [24,27] cited the 3C Collaboration Model and the Collaboration Space Model. Originally modified by Fuks et al [15] in 2008, the 3C Collaboration Model addresses aspects of communication, coordination, cooperation, and collaboration in the context of developing groupware applications (such as chat platforms). The Collaboration Space Model was designed to help researchers study technology for collaboration purposes in health care [14].

Most studies providing a definition were published in journals in the subject area of medicine (n=29) [22,24,25,27-44,46-50,52-54], with the main subject categories including health informatics (n=12) [22,25,27,30,32,33,40,43,47,49,50,53], health policy (n=5) [28,35,36,38,44], and multidisciplinary (n=4) [29,31,52,54] (Figure 3B). Notably, relative to the number of papers in medical specialties, few papers in this category provided a definition. Approximately every fifth literature review provided a definition, in contrast to every 24th study using quantitative research methods. The provision of definitions across settings was relatively even, except for papers classified as “others,” which defined more concepts. Only one in 65 studies about eConsults or eReferrals provided a definition.

The following paragraphs summarize key aspects extracted from definitions provided in the papers and references. Definitions of technology, such as mHealth, telehealth, and health information exchange, were analyzed together to inductively identify common themes and dimensions. An overview of these aspects, along with their references, is provided in Multimedia Appendix 4.

### Digital Support

The definitions acknowledged a broad variety of digital tools, ranging from smartphone apps to desktop-based communication systems, applied to various health care contexts and settings. A key requisite for the effective use of technological tools is a good technological infrastructure, including standards and interoperability, particularly concerning the possibility of different tools sharing and using accurate data in compliance with data protection laws. Digital tools are recognized for improving the accessibility of health care services and patient data through prompt and, in some cases, real-time exchange of data across geographical and institutional boundaries, providing information “whenever and wherever needed” [43]. Some definitions suggest that digital tools induce “global thinking” [26,55,56] among users. Furthermore, these digital tools create a “networked” [26] environment and lead to an “expansion and cultural transformation of traditional health care” [39]. The primary objective of using digital tools in health care is to enhance patient care by providing “the right care in the right place at the right time” [57]. Definitions focus on the empowerment aspect of digital tools by “making the knowledge bases (…) accessible” [58] and simultaneously stress the enhanced efficiency by “avoiding duplicative and unnecessary diagnostic or therapeutic interventions” [58]. Despite the possibilities of digital tools, Eysenbach [58] emphasized that care must be taken to address the digital divide and ensure that all people can benefit from the technology used.

### Communication

Communication is defined as the exchange of information in a two-way interactive process between the sender and the receiver. Sharma et al [59] specifies that communication, according to Lasswell’s communication theory, is “who says what to whom in what channel with what effect.” Essential to all communication processes is an established common ground, sender and receiver must use a common “system of symbols, signs, or behavior” [7] to ensure that information is conveyed in a “meaningful way” [23] and understood correctly. To achieve high-quality and effective communication, four dimensions are highlighted: (1) openness, characterized by the ability to express information “without fear of repercussions or misunderstanding” [22,60], (2) accuracy of the information and message, (3) timeliness of the information exchange, as delays might lead to redundant work, and (4) satisfaction with the communication. The goal of communication extends beyond mere information exchange. Its purpose is to elicit an “effect” [23,61], such as specific action, an improved understanding of a patient’s health status or to “establish and maintain relationships” [14].

### Coordination

Coordination is described as the management of individuals, activities, and resources, based on mutual respect and shared values. Specific to coordination is that tasks performed by separate agents are interdependent, requiring a constant update of “mutual knowledge, mutual beliefs, and mutual assumptions […] moment by moment” [62] and structured management to jointly achieve goals. In health care, the aim of coordination activities is to integrate care processes to ensure “appropriate delivery of health care services” [28] and a continuity of care across “all of a patient’s conditions, needs, clinicians, and settings” [29,63].

### Cooperation

Cooperation is characterized as “multiple individuals working together in a conscious way” [64], entailing the conscious integration of tasks, knowledge, and skills among individuals. The agents strive toward a shared goal and are motivated to work together as a team. Tasks are not merely interconnected but intentionally distributed among the participants, who often share a common workspace. The distribution of tasks means that each person “has only a partial vision of the entire situation” [32]. The tasks are subsequently amalgamated to contribute toward the shared goal: problem-solving and decision-making, incorporating the diverse views and competencies of all those involved.

### Collaboration

Collaboration is portrayed as “collective action” [65] by interprofessional health care workers. Engaging in such collective action requires true partnership based on respect, mutual recognition of, and trust in one another’s abilities, knowledge, and skills. These requisites enable agents to “pool and share” [66] planning and decision-making, responsibilities, and challenges. Collaboration is characterized by (1) shared power (based on professional equality and a pronounced understanding of professional roles), (2) organizational factors (such as administrative and organizational support for collaboration, clearly defined organizational structures, a shared budget, an open and respectful environment, a culture and mindset facilitating collaboration, and established mechanisms to deal with conflicts), (3) team characteristics (including the size and composition of the team and clear leadership), and (4) individual characteristics (such as the willingness, time, and resources to engage in collaborative efforts, the individuals’ age, gender, and educational background). The aim of collaboration is to collectively address “the complexities of patient needs” [26] to provide the highest quality of care.

### Synthesis

**Figure 4:**
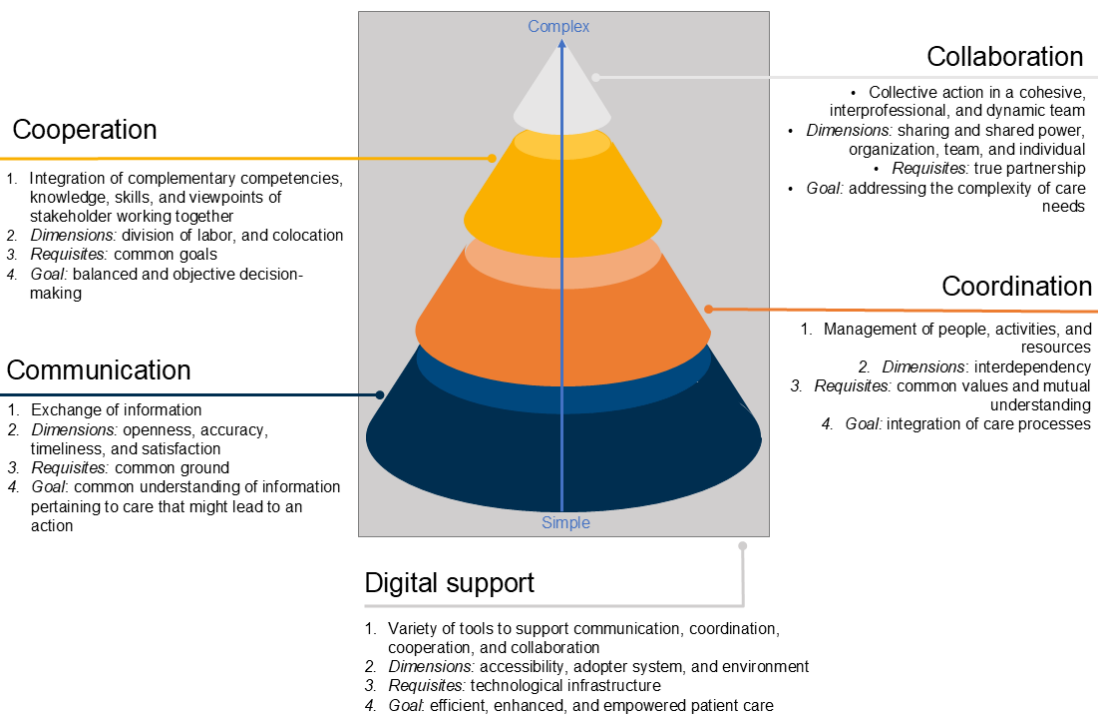
Main attributes of digitally supported communication, coordination, cooperation, and collaboration, as defined in scientific records extracted through a scoping review and their corresponding sources.

Communication, coordination, cooperation, and collaboration are interrelated concepts that progressively build upon each other (Figure 4). At the base of this conceptual framework is communication, which allows for an exchange of information. This enables the coordination of people, activities, and resources. Coordination, in turn, lays the groundwork for cooperation, which involves the division of labor and subsequent unification to achieve a shared goal. Ultimately, cooperation fosters collaboration in which agents collectively address complex care needs. While exchanging information is a rather simple task, achieving effective collaboration is a complex undertaking. All elements of this framework can be supported by a variety of digital tools.

## Discussion

### Principal Findings

This scoping review identified 407 papers addressing interprofessional D4C in health care. While all papers portray D4C, only a small fraction define the digital concept (25/407; 6.1%) or the mode of interaction supported by the digital tool (27/407, 6.6%). Even fewer provide definitions backed by a reference. In most papers, the digital tool itself and its integration into the context are portrayed and evaluated. The scarcity of definitions within the literature underscores a reliance on implicit understandings of D4C concepts in the health care sector.

Given the relative void of explicit D4C definitions in papers, the surge of D4C tools in recent years and their challenging integration into standard practice, we developed a comprehensive D4C framework by analyzing the extracted definitions from the papers and their references. The D4C framework is structured as a pyramid, with communication as the foundational layer for coordination, which enables cooperation, positioning collaboration at the apex. This hierarchy, although reflecting a common understanding of the concepts, is not uncontested. Some authors debate the order of cooperation and coordination, or that of coordination and collaboration, proposing that collaboration might enable coordinated care [30,67,68]. For instance, Fuks et al [15] define collaboration as the “interplay between communication, coordination, and cooperation.” Others view collaboration as a spectrum, ranging from mere information exchange to complex interactional efforts, as understood by collaboration in this paper [34,36,37,69,70]. The concept of collaboration remains thus one of the most debated concepts [14,26,65,70,71]. This suggests that even though communication, coordination, cooperation, and collaboration are depicted as separate concepts in our framework, their boundaries are fluid and intertwined.

The effectiveness of each layer within the D4C framework depends on the extent to which the dimensions (such as openness, accuracy, timeliness, and satisfaction for communication) and the requisites (such as common values and mutual understanding for coordination) are fulfilled. It further depends on external factors, including the political conditions and the overarching context in which the D4C tools are deployed, which influence the dynamics and outcomes of D4C processes [72].

The lack of clear definitions within the literature currently presents a challenge to operationalization, that is, the efficient, transparent, and standardized evaluation and comparison of D4C tools. The development of this D4C framework was driven by the need to address differing implicit definitions. While many studies identified in the review described the technology exhaustively, they lacked an equally exhaustive analysis of the underlying modes of interaction that the technology was supposed to support. The value of our framework lies in its capacity to structure concepts pertaining to D4C, allowing for improved stakeholder engagement and possibly enabling operationalization of these concepts [73,74].

As such, the proposed D4C framework can inform technology suppliers and policy makers, research, and practice, fostering a comprehensive understanding of D4C definitions used in a given project and the intended interaction enabled through a specific D4C tool. Such clarity could guide technology providers in developing adequate tools for the intended interaction, be it communication, coordination, cooperation, or collaboration. Policy makers can use the framework to formulate or revise guidelines and regulations that support effective integration of D4C tools into health care settings, ensuring that they contribute to improved interprofessional interaction and workflow, possibly enhancing patient care.

### Limitations

Despite our comprehensive search strategy, which included databases from different disciplines such as health sciences, social sciences, and life sciences, we may not have identified all relevant literature as technological databases such as IEEE Xplore and ACM Digital Library were not searched. To mitigate this limitation, we conducted a manual search of the references cited in the included papers. Although we believe that the identified studies provide a robust foundation for the D4C framework, future research should aim to include other sources such as gray literature and industry reports. Another limitation is that we did not perform a comprehensive search of interprofessional interaction without digital tools and deliberately only included explicit definitions of D4C concepts. While a more in-depth analysis of implicit definitions could have provided a more nuanced D4C framework, only including explicit definitions showcases how authors understand the concepts and underscore their importance. Basing the D4C framework on definitions included or referenced in the literature further enhances the likelihood of successfully translating the conceptual framework into practical application.

### Conclusion

Our review is the first to present a comprehensive D4C framework derived from scientific literature. By providing a structured approach to D4C tools and the supported communication, coordination, cooperation, and collaboration, our framework can assist stakeholders in their understanding of D4C tools and guide development and deployment. Although the framework builds a solid foundation, additional research is needed to further operationalize the D4C (framework) and to establish a maturity model to efficiently measure the impact of D4C tools across diverse health care settings.

## Appendix

Multimedia Appendix 1 PRISMA checklist.

Multimedia Appendix 2 Search strategies.

Multimedia Appendix 3 In-depth details about included articles.

Multimedia Appendix 4 Attributes of digitally supported communication, coordination, cooperation and collaboration as defined through the scoping review and their corresponding sources.

## Abbreviations

D4C: digital tools for communication, coordination, cooperation, and collaboration
mHealth: mobile health
PRISMA: Preferred Reporting Items for Systematic Reviews and Meta-Analyses
PRISMA-ScR: Preferred Reporting Items for Systematic Reviews and Meta-Analyses extension for Scoping Reviews

## Data Availability

The datasets generated or analyzed during this study are available from the corresponding author on reasonable request.
