# Supplementary material for: Toward a Conceptual Framework for Digitally Supported Communication, Coordination, Cooperation, and Collaboration in Interprofessional Health Care: Scoping Review
Source: J Med Internet Res. 2025 May 26;27:e69276. doi: 10.2196/69276 (PMC12149778; doi:10.2196/69276)
Supplement: Multimedia Appendix 4 [file jmir_v27i1e69276_app4.docx]

|  | Digital supported | Communication | Coordination | Cooperation | Collaboration |
| --- | --- | --- | --- | --- | --- |
| Definition | - Variety of digital tools applied in different fields and settings [1–42] | - Exchange of information [17,43–57] - Two-way interaction [17,50,54–58] | - Management of people, activities and resources [5,45,48,52,54,59–66] | - Integration of complementary competencies, knowledge, skills and viewpoints of stakeholder [52,67,68] | - Collective action in a cohesive, interprofessional and dynamic team [5,17,48,55,62,69–80] |
| Dimension | - Accessibility [3,4,10,13,23–27,30,34–41,81–83] - Adopter system [7,10–12,17] - Environment [1,3,11–13,17,25,29,39,82] | - Openness [43,50,63,65,73] - Accuracy [43,46,49,50,56,63,65,84] - Timeliness [17,43,49,50,63,65,73] - Satisfaction [65,73,84] | - Interdependency [52,54,62,63,66] | - Division of labour [59,66,67,75,85] - Co-location [5,45,48,52] | - Sharing and shared power [17,55,62,75,79,80,86–90] - Structural factors [53,69,70,72,75,89–92] - Team-based factors [55,72,89,90] - Individual factors [53,55,72,75,80,87,89,90,93,94] |
| Requisites | - Technological infrastructure [14,21,32,38,41,81,83,95,96] | - Common ground [17,47,49,56,57,64,97] | - Common values and mutual understanding [50,64,66,98] | - Common goals [46,50,68] | - True partnership[17,55,62,68–71,73,74,79,80,86,87,90–93,98] |
| Goal | - Efficient, enhanced and empowered patient-care [1–3,6,7,10,13–16,23,29,30,35,36,38,41,42,95,96] | - Common understanding of information pertaining to care that might lead to an action [17,45,48,49,51,58,63,65,97] - Relationship building and maintaining [48,49,51,56,57] | - Integration of care processes [46,50,54,60,62,63,99,100] | - Balanced and objective decision-making [68] | - Addressing the complexity of care needs [5,17,55,73,76,78–80,89] |

**Appendix 4**. Attributes of digitally supported communication, coordination, cooperation and collaboration as defined through the scoping review and their corresponding sources

References

1. Mills J, Fox J, Damarell R, Tieman J, Yates P. Palliative care providers’ use of digital health and perspectives on technological innovation: a national study. BMC Palliat Care 2021;20(1). doi:10.1186/s12904-021-00822-2

2. World Health Organization. Global strategy on digital health 2020-2025; 2021. ISBN:978-92-4-002093-1.

3. Meskó B, Drobni Z, Bényei É, Gergely B, Győrffy Z. Digital health is a cultural transformation of traditional healthcare. Mhealth 2017;3:38. PMID:29184890

4. Alkmim MBM, Marcolino MS, Figueira RM, Sousa L, Nunes MS, Cardoso CS, Ribeiro AL. Factors associated with the use of a teleconsultation system in Brazilian primary care. Telemed J E Health 2015;21(6):473-483. PMID:25785650

5. Husain A, Cohen E, Dubrowski R, Jamieson T, Kurahashi AM, Lokuge B, Rapoport A, Saunders S, Stasiulis E, Stinson J, Subramaniam S, Wegier P, Barwick M. A Clinical Communication Tool (Loop) for Team-Based Care in Pediatric and Adult Care Settings: Hybrid Mixed Methods Implementation Study. J Med Internet Res 2021;23(3):e25505. PMID:33656445

6. Johansson AM, Lindberg I, Söderberg S. The views of health-care personnel about video consultation prior to implementation in primary health care in rural areas. Prim Health Care Res Dev 2014;15(2):170-179. PMID:23402617

7. Tran V, Lam MK, Amon KL, Brunner M, Hines M, Penman M, Lowe R, Togher L. Interdisciplinary eHealth for the care of people living with traumatic brain injury: A systematic review. Brain Inj 2017;31(13-14):1701-1710. PMID:29064300

8. Glasgow RE, Phillips SM, Sanchez MA. Implementation science approaches for integrating eHealth research into practice and policy. Int J Med Inform 2014;83(7):e1-11. PMID:23910896

9. Ross J, Stevenson F, Lau R, Murray E. Factors that influence the implementation of e-health: a systematic review of systematic reviews (an update). Implement Sci 2016;11(1):146. PMID:27782832

10. Eysenbach G. What is e-health? J Med Internet Res 2001;3(2):E20. PMID:11720962

11. Cashen MS, Dykes P, Gerber B. eHealth technology and Internet resources: barriers for vulnerable populations. Journal of Cardiovascular Nursing 2004;19(3):209-214. doi:10.1097/00005082-200405000-00010

12. Boogerd EA, Arts T, Engelen LJ, van de Belt TH. "What Is eHealth": Time for An Update? JMIR Res Protoc 2015;4(1):e29. PMID:25768939

13. Pendergrass JC, Chandrasekaran R. Key Factors Affecting Ambulatory Care Providers' Electronic Exchange of Health Information With Affiliated and Unaffiliated Partners: Web-Based Survey Study. JMIR Med Inform 2019;7(4):e12000. PMID:31697241

14. Akhlaq A, Sheikh A, Pagliari C. Defining Health Information Exchange: Scoping Review of Published Definitions. J Innov Health Inform 2017;23(4):838. PMID:28346130

15. Rocha GA, Silva RKdSe, Neto FJdC, Fontes JH, Nascimento JMFd, Bastos SNMAN. Comunicação efetiva para segurança do paciente e o uso de tecnologias da informação em saúde. Rev. Enferm. Atual In Derme 2020;93(31). doi:10.31011/reaid-2020-v.93-n.31-art.712

16. Brown-Manhertz D. Using smartphones to improve interdisciplinary collaboration. Nurse Pract 2017;42(4):1-5. PMID:28306663

17. Palanisamy R, Taskin N, Verville J. Impact of Trust and Technology on Interprofessional Collaboration in Healthcare Settings. International Journal of e-Collaboration 2017;13(2):10-44. doi:10.4018/IJeC.2017040102

18. Bordé A, Fromm C, Kapadia F, Molla DS, Sherwood E, Brandt Serensen J. GAID White Paper On ICT4D Health. New York; 2010.

19. Ganasegeran K, Renganathan P, Rashid A, Al-Dubai SAR. The m-Health revolution: Exploring perceived benefits of WhatsApp use in clinical practice. Int J Med Inform 2017;97:145-151. PMID:27919374

20. Nam HS, Park E, Heo JH. Facilitating Stroke Management using Modern Information Technology. J Stroke 2013;15(3):135-143. PMID:24396807

21. World Health Organization. mHealth: New horizons for health through mobile technologies. 3rd ed. Geneva; 2011. Global Observatory for eHealth series.

22. Turner K, Bobonis Babilonia M, Naso C, Nguyen O, Gonzalez BD, Oswald LB, Robinson E, Elston Lafata J, Ferguson RJ, Alishahi Tabriz A, Patel KB, Hallanger-Johnson J, Aldawoodi N, Hong Y-R, Jim HSL, Spiess PE. Health Care Providers' and Professionals' Experiences With Telehealth Oncology Implementation During the COVID-19 Pandemic: A Qualitative Study. J Med Internet Res 2022;24(1):e29635. PMID:34907900

23. Jiang CY, El-Kouri NT, Elliot D, Shields J, Caram MEV, Frankel TL, Ramnath N, Passero VA. Telehealth for Cancer Care in Veterans: Opportunities and Challenges Revealed by COVID. JCO Oncol Pract 2021;17(1):22-29. PMID:32970512

24. Chaet D, Clearfield R, Sabin JE, Skimming K. Ethical practice in Telehealth and Telemedicine. J Gen Intern Med 2017;32(10):1136-1140. PMID:28653233

25. Giordano V, Koch H, Godoy-Santos A, Dias Belangero W, Esteves Santos Pires R, Labronici P. WhatsApp Messenger as an Adjunctive Tool for Telemedicine: An Overview. Interact J Med Res 2017;6(2):e11. PMID:28733273

26. Mohammad Reza F. Aghdam, Aleksandar Vodovnik, Rania Adel Hameed. 14th European Congress on Digital Pathology. Journal of Pathology Informatics 2019;10(1):32. doi:10.4103/2153-3539.270744

27. Silsand L, Severinsen G-H, Berntsen G. Preservation of Person-Centered Care Through Videoconferencing for Patient Follow-up During the COVID-19 Pandemic: Case Study of a Multidisciplinary Care Team. JMIR Form Res 2021;5(3):e25220. PMID:33646965

28. Bashshur, Rashid, L. Telemedicine effects: Cost, quality, and access. Journal of Medical Systems 1995;19:81-91.

29. Demiris G, Edison K, Vijaykumar S. A comparison of communication models of traditional and video-mediated health care delivery. Int J Med Inform 2005;74(10):851-856. PMID:16005258

30. Sood S, Mbarika V, Jugoo S, Dookhy R, Doarn CR, Prakash N, Merrell RC. What is telemedicine? A collection of 104 peer-reviewed perspectives and theoretical underpinnings. Telemed J E Health 2007;13(5):573-590. PMID:17999619

31. Eron L. Telemedicine: the future of outpatient therapy? Clin Infect Dis 2010;51 Suppl 2:S224-30. PMID:20731581

32. Shah T, Bhatt CM. Telemedicine-The New Era of Healthcare. CSI Communications 2013;16.

33. Tensen E, van der Heijden JP, Jaspers MWM, Witkamp L. Two Decades of Teledermatology: Current Status and Integration in National Healthcare Systems. Curr Dermatol Rep 2016;5:96-104. PMID:27182461

34. World Health Organization. A Health Telematics Policy in support of WHO's Health-for-All Strategy for Global Health Development: Report of the WHO Group Consultation on Health Telematics. Geneva; 1998.

35. Voran D. Telemedicine and beyond. Mo Med 2015;112(2):129-135. PMID:25958658

36. Alami H, Gagnon MP, Wootton R, Fortin JP, Zanaboni P. Exploring factors associated with the uneven utilization of telemedicine in Norway: a mixed methods study. BMC Med Inform Decis Mak 2017;17(1):180. PMID:29282048

37. Hong Z, Li N, Li D, Li J, Li B, Xiong W, Lu L, Li W, Zhou D. Telemedicine During the COVID-19 Pandemic: Experiences From Western China. J Med Internet Res 2020;22(5):e19577. PMID:32349962

38. Klonoff DC. Using Telemedicine to Improve Outcomes in Diabetes—An Emerging Technology. Journal of Diabetes Science and Technology 2009;3(4):624-628.

39. Wootton R, Bonnardot L. In what circumstances is telemedicine appropriate in the developing world? JRSM Short Rep 2010;1(5):37. PMID:21103129

40. Lonergan PE, Washington Iii SL, Branagan L, Gleason N, Pruthi RS, Carroll PR, Odisho AY. Rapid Utilization of Telehealth in a Comprehensive Cancer Center as a Response to COVID-19: Cross-Sectional Analysis. J Med Internet Res 2020;22(7):e19322. PMID:32568721

41. Pan American Health Organization, World Health Organization. Strategy and Plan of the Action on eHealth: 51st Directing Councal. 63rd Session of the Regional Committee. Washington; 2011.

42. World Health Organization. eHealth 2023 URL: https://www.emro.who.int/health-topics/ehealth/ [accessed 2023-12-12].

43. Hoonakker PLT, Carayon P, Walker JM, Brown RL, Cartmill RS. The effects of Computerized Provider Order Entry implementation on communication in Intensive Care Units. Int J Med Inform 2013;82(5):e107-17. PMID:23298435

44. Dalkin S, Lhussier M, Jones D, Phillipson P, Cunningham W. Open communication strategies between a triad of 'experts' facilitates death in usual place of residence: A realist evaluation. Palliat Med 2018;32(5):980-989. PMID:29400631

45. Sheehan J, Laver K, Bhopti A, Rahja M, Usherwood T, Clemson L, Lannin NA. Methods and Effectiveness of Communication Between Hospital Allied Health and Primary Care Practitioners: A Systematic Narrative Review. J Multidiscip Healthc 2021;14:493-511. PMID:33654406

46. Tang T, Heidebrecht C, Coburn A, Mansfield E, Roberto E, Lucez E, Lim ME, Reid R, Quan SD. Using an electronic tool to improve teamwork and interprofessional communication to meet the needs of complex hospitalized patients: A mixed methods study. Int J Med Inform 2019;127:35-42. PMID:31128830

47. Merriam-Webster. communication URL: https://www.merriam-webster.com/dictionary/communication [accessed 2023-12-12].

48. Eikey EV, Reddy MC, Kuziemsky CE. Examining the role of collaboration in studies of health information technologies in biomedical informatics: A systematic review of 25 years of research. J Biomed Inform 2015;57:263-277. PMID:26264406

49. Ash JS, Berg M, Coiera E. Some unintended consequences of information technology in health care: the nature of patient care information system-related errors. J Am Med Inform Assoc 2004;11(2):104-112. PMID:14633936

50. Salas E, Wilson KA, Murphy CE, King H, Salisbury M. Communicating, coordinating, and cooperating when lives depend on it: tips for teamwork. Jt Comm J Qual Patient Saf 2008;34(6):333-341. PMID:18595379

51. Sharma G, Qiang Y, Wenjun S, Qi L. Communication in virtual world: Second life and business opportunities. Inf Syst Front 2013;15(4):677-694. doi:10.1007/s10796-012-9347-z

52. Fuks H, Raposo A, Gerosa MA, Pimental M, Lucena CJP. The 3C Collaboration Model. In: Kock N, editor. Encyclopedia of E-Collaboration: IGI Global; 2008. ISBN:9781599040004. p. 637–644.

53. Youngwerth J, Twaddle M. Cultures of interdisciplinary teams: how to foster good dynamics. J Palliat Med 2011;14(5):650-654. PMID:21476853

54. Abraham J, Kannampallil TG, Patel VL. Bridging gaps in handoffs: a continuity of care based approach. J Biomed Inform 2012;45(2):240-254. PMID:22094355

55. Fewster-Thuente L, Velsor-Friedrich B. Interdisciplinary collaboration for healthcare professionals. Nurs Adm Q 2008;32(1):40-48. PMID:18160862

56. Alshatnawi EAR. Assessing Communication Skills among Jordanian Tour Guides: German Tourists Perceptions. Journal of Management Research 2013;6(1):1. doi:10.5296/jmr.v6i1.4361

57. Keyton J. Communication & organizational culture: A key to understanding work experiences. 2. ed. Los Angeles, Calif.: Sage; 2011. ISBN:9781412980227.

58. Thomas SK, Coleman JJ. The impact of computerised physician order entry with integrated clinical decision support on pharmacist–physician communication in the hospital setting: a systematic review of the literature. Eur J Hosp Pharm 2012;19(4):349-354. doi:10.1136/ejhpharm-2012-000110

59. Iversen TB, Melby L, Toussaint P. Instant messaging at the hospital: supporting articulation work? Int J Med Inform 2013;82(9):753-761. PMID:23746431

60. La Rocca A, Hoholm T. Coordination between primary and secondary care: the role of electronic messages and economic incentives. BMC Health Serv Res 2017;17(1):149. PMID:28212653

61. Craig C, Eby D, Whittington J. Care Coordination Model: Better Care at Lower Cost for People with Multiple Health and Social Needs. Innovation Series 2011.

62. McDonald KM, Sundaram V, Bravata DM, Lewis R, Lin N, Kraft SA, McKinnon M, Paguntalan H, Owens DK. Closing the Quality Gap: A Critical Analysis of Quality Improvement Strategies (Vol. 7: Care Coordination) 2007. PMID:20734531

63. Shortell SM, Zimmerman JE, Rousseau DM, Gillies RR, Wagner DP, Draper EA, Knaus WA, Duffy J. The performance of intensive care units: does good management make a difference? Med Care 1994;32(5):508-525. PMID:8182978

64. Resnick LB, Levine JM, Teasley SD, editors. Grounding in Communication (Chapter 7). Washington: APA Books; 1991.

65. Shortell SM, Rousseau DM, Gillies RR, Devers KJ, Simons TL. shortell1991 // Organizational assessment in intensive care units (ICUs): construct development, reliability, and validity of the ICU nurse-physician questionnaire. Med Care 1991;29(8):709-726. PMID:1875739

66. Schmidt K, Simonee C. Coordination mechanisms: Towards a conceptual foundation of CSCW systems design. Computer Supported Cooperative Work (CSCW) 1996;5:155-200.

67. Pelayo S, Anceaux F, Rogalski J, Elkin P, Beuscart-Zephir M-C. A comparison of the impact of CPOE implementation and organizational determinants on doctor-nurse communications and cooperation. Int J Med Inform 2013;82(12):e321-30. PMID:22999779

68. Flemming D, Hübner U. How to improve change of shift handovers and collaborative grounding and what role does the electronic patient record system play? Results of a systematic literature review. Int J Med Inform 2013;82(7):580-592. PMID:23628146

69. Bjørkquist C, Forss M, Samuelsen F. Collaborative challenges in the use of telecare. Scand J Caring Sci 2019;33(1):93-101. PMID:30113071

70. Vos JFJ, Boonstra A, Kooistra A, Seelen M, van Offenbeek M. The influence of electronic health record use on collaboration among medical specialties. BMC Health Serv Res 2020;20(1):676. PMID:32698807

71. Kuziemsky CE, Varpio L. A model of awareness to enhance our understanding of interprofessional collaborative care delivery and health information system design to support it. Int J Med Inform 2011;80(8):e150-60. PMID:21317027

72. Careau E, Vincent C, Swaine BR. Consensus group session of experts to describe interprofessional collaboration processes in team meetings. J Interprof Care 2011;25(4):299-301. PMID:21554062

73. Hansen HE, Biros MH, Delaney NM, Schug VL. Research utilization and interdisciplinary collaboration in emergency care. Acad Emerg Med 1999;6(4):271-279. PMID:10230977

74. Broers T, Poth C, Medves J. What’s in a Word? Understanding “Interprofessional Collaboration" from the Student's Perspective. Journal of Research in Interprofessional Practice and Education 2009;1(1).

75. D'Amour D. Structuration de la collaboration Structuration de la collaboration interprofessionnelle dans les services de santé de première ligne au Québec. Ottawa: National Library of Canada; 1997.

76. World Health Organization. Framework for Action on Interprofessional Education & Collaborative Practice; 2010.

77. Houldin AD, Naylor MD, Haller DG. Physician-nurse collaboration in research in the 21st century. J Clin Oncol 2004;22(5):774-776. PMID:14990631

78. Dey RM, Vries MJW de, Bosnic-Anticevich S. Collaboration in chronic care: unpacking the relationship of pharmacists and general medical practitioners in primary care. Int J Pharm Pract 2011;19(1):21-29. PMID:21235656

79. McDonald C, McCallin A. Interprofessional collaboration in palliative nursing: what is the patient-family role? International Journal of Palliative Nursing 2010;16(6):286-289. PMID:20925291

80. D'Amour D, Ferrada-Videla M, San Martin Rodriguez L, Beaulieu M-D. The conceptual basis for interprofessional collaboration: core concepts and theoretical frameworks. J Interprof Care 2005;19 Suppl 1:116-131. PMID:16096150

81. Souza CHA de, Morbeck RA, Steinman M, Hors CP, Bracco MM, Kozasa EH, Leão ER. Barriers and Benefits in Telemedicine Arising Between a High-Technology Hospital Service Provider and Remote Public Healthcare Units: A Qualitative Study in Brazil. Telemed J E Health 2017;23(6):527-532. PMID:27911667

82. Zachariah R, Bienvenue B, Ayada L, Manzi M, Maalim A, Engy E, Jemmy JP, Ibrahim Said A, Hassan A, Abdulrahaman F, Abdulrahman O, Bseiso J, Amin H, Michalski D, Oberreit J, Draguez B, Stokes C, Reid T, Harries AD. Practicing medicine without borders: tele-consultations and tele-mentoring for improving paediatric care in a conflict setting in Somalia? Trop Med Int Health 2012;17(9):1156-1162. PMID:22845678

83. Zanaboni P, Wootton R. Adoption of routine telemedicine in Norwegian hospitals: progress over 5 years. BMC Health Serv Res 2016;16:496. PMID:27644324

84. Roberts KH, O'Reilly CA. Measuring organizational communication. Journal of Applied Psychology 1974;59(3):321-326. doi:10.1037/h0036660

85. Schmidt K. Cooperative Work and its Articulation: Requirements for Computer Support. Le Travail Humain 1993;57(4):345-366.

86. Zelko E, Ramsak Pajk J, Škvarč NK. An Innovative Approach for Improving Information Exchange between Palliative Care Providers in Slovenian Primary Health-A Qualitative Analysis of Testing a New Tool. Healthcare (Basel) 2022;10(2). PMID:35206829

87. Zillich AJ, McDonough RP, Carter BL, Doucette WR. Influential characteristics of physician/pharmacist collaborative relationships. Ann Pharmacother 2004;38(5):764-770. PMID:15031418

88. Hertzum M. Collaborative information seeking: The combined activity of information seeking and collaborative grounding. Information Processing & Management 2008;44(2):957-962. doi:10.1016/j.ipm.2007.03.007

89. D'Amour D, Sicotte C, Lévy R. L'action collective au sein d'équipes interprofessionnelles dans les services de santé. Sciences sociales et santé 1999;17(3):67-94. doi:10.3406/sosan.1999.1468

90. Schmied V, Mills A, Kruske S, Kemp L, Fowler C, Homer C. The nature and impact of collaboration and integrated service delivery for pregnant women, children and families. J Clin Nurs 2010;19(23-24):3516-3526. PMID:20946442

91. Weissenborn M, Haefeli WE, Peters-Klimm F, Seidling HM. Interprofessional communication between community pharmacists and general practitioners: a qualitative study. Int J Clin Pharm 2017;39(3):495-506. PMID:28315115

92. Andersson J, Ahgren B, Axelsson SB, Eriksson A, Axelsson R. Organizational approaches to collaboration in vocational rehabilitation-an international literature review. International Journal of Integrated Care 2011;11:e137. PMID:22128280

93. Arslanian-Engoren CM. Lived experiences of CNSs who collaborate with physicians: a phenomenological study. Clin Nurse Spec 1995;9(2):68-74. PMID:7600484

94. Snyder ME, Zillich AJ, Primack BA, Rice KR, Somma McGivney MA, Pringle JL, Smith RB. Exploring successful community pharmacist-physician collaborative working relationships using mixed methods. Res Social Adm Pharm 2010;6(4):307-323. PMID:21111388

95. Watkinson F, Dharmayat KI, Mastellos N. A mixed-method service evaluation of health information exchange in England: technology acceptance and barriers and facilitators to adoption. BMC Health Serv Res 2021;21(1):737. PMID:34303379

96. Kuperman GJ. Health-information exchange: why are we doing it, and what are we doing? J Am Med Inform Assoc 2011;18(5):678-682. PMID:21676940

97. Lingard L, Reznick R, Espin S, Regehr G, DeVito I. Team communications in the operating room: talk patterns, sites of tension, and implications for novices. Acad Med 2002;77(3):232-237. PMID:11891163

98. Hawley G, Hepworth J, Jackson C, Wilkinson SA. Integrated care among healthcare providers in shared maternity care: what is the role of paper and electronic health records? Aust J Prim Health 2017;23(4):397-406. PMID:28606289

99. Walsh C, Siegler EL, Cheston E, O'Donnell H, Collins S, Stein D, Vawdrey DK, Stetson PD. Provider-to-provider electronic communication in the era of meaningful use: a review of the evidence. J Hosp Med 2013;8(10):589-597. PMID:24101544

100. O'Malley AS, Grossman JM, Cohen GR, Kemper NM, Pham HH. Are electronic medical records helpful for care coordination? Experiences of physician practices. J Gen Intern Med 2010;25(3):177-185. PMID:20033621
